# Lipid-based nanoparticles and RNA as innovative neuro-therapeutics

**DOI:** 10.3389/fphar.2022.900610

**Published:** 2022-08-09

**Authors:** Maria Tsakiri, Cristina Zivko, Costas Demetzos, Vasiliki Mahairaki

**Affiliations:** ^1^ Section of Pharmaceutical Technology, Department of Pharmacy, School of Health Sciences, National and Kapodistrian University of Athens, Athens, Greece; ^2^ Department of Genetic Medicine, Johns Hopkins School of Medicine, Baltimore, MD, United States; ^3^ The Richman Family Precision Medicine Center of Excellence in Alzheimer’s Disease, Johns Hopkins Medicine, Baltimore, MD, United States

**Keywords:** lipid nanoparticles, liposomes, lipoplexes, solid lipid nanoparticles, gene delivery, RNA, central nervous system

## Abstract

RNA-delivery is a promising tool to develop therapies for difficult to treat diseases such as neurological disorders, by silencing pathological genes or expressing therapeutic proteins. However, in many cases RNA delivery requires a vesicle that could effectively protect the molecule from bio-degradation, bypass barriers i.e., the blood brain barrier, transfer it to a targeted tissue and efficiently release the RNA inside the cells. Many vesicles such as viral vectors, and polymeric nanoparticles have been mentioned in literature. In this review, we focus in the discussion of lipid-based advanced RNA-delivery platforms. Liposomes and lipoplexes, solid lipid nanoparticles and lipid nanoparticles are the main categories of lipidic platforms for RNA-delivery to the central nervous systems (CNS). A variety of surface particles’ modifications and routes of administration have been studied to target CNS providing encouraging results *in vivo*. It is concluded that lipid-based nanoplatforms will play a key role in the development of RNA neuro-therapies.

## Introduction

Approximately 50 years ago, the first conversation of whether gene therapy could be the solution for the treatment of serious diseases such as cancer or genetic disorders began (Friedmann and Roblin, 1972). Since then, the science of gene therapy has flourished. However, currently, only a few products based on *in vivo* or *ex vivo* gene therapy have been approved by the US Food and Drug Administration (FDA) and the European Medicines Agency (EMA) as serious limitations have not yet been overcome. Indeed, the first gene therapy clinical trials resulted in serious adverse effects or even deaths that lead the US National Institute of Health (NIH) to suspend the trials in pre-clinical stages (Orkin and Motulsky, 1996).

Although today many of the early shortcomings of this technology such as off-target effects, vector safety, or inefficient administration have been solved, there still remain some unsolved questions. Especially in the case of *in vivo* gene therapy, nucleic acid should bypass the immune system and bio-degradation processes of the human organism, reach the target tissue, and even cross cell or nucleus membranes. Thus, the accomplishment of the therapeutic genetic modifications requires that the nucleic acid will remain effective till the time of its expression by the target cells. For this purpose, the use of a vector is very common. The vector is responsible for the delivery of the nucleic acid to the target, avoiding its degradation and systematic adverse effects. The majority of vectors are viral vectors such as adenoviruses (Zhu et al., 2020; Voysey et al., 2021; Watanabe et al., 2021), retroviruses (Rastegar et al., 2009), lentiviruses (Maude et al., 2014; Sessa et al., 2016) or adeno-associated viruses (Wuh-Liang et al., 2012; Keiser et al., 2016; Mendell et al., 2017; Wang et al., 2018). Currently, many scientific groups propose generally safe viral vectors as efficient gene delivery platforms (Walther and Stein, 2000; Bulcha et al., 2021; Selvaraj et al., 2021).

Nevertheless, other alternatives to the viral vectors have also been developed and studied over a period of approximately 40 years (Patil et al., 2019; Faneca, 2021). Indeed, the first medicinal product that used a lipid nanoplatform (LNP) for the delivery of a siRNA molecule has been approved by FDA and EMA in 2018 by the trade name Patisiran or Onppatro respectively for the treatment of hereditary transthyretin-mediated amyloidosis (hATTR amyloidosis) in adult patients with stage 1 or stage 2 polyneuropathy (DeWeerdt, 2019). Today, this technology has been used for the development of mRNA COVID-19 vaccines (Tsakiri et al., 2021), while a few other similar platforms are in clinical trials (Damase et al., 2021).

Diseases of the central nervous system (CNS) such as tumors or neurodegenerative pathologies, have high mortality rates as there are no medicinal products to cure them (Liang et al., 2021). For example, approximately 6.2 million American citizens aged ≥65 years were estimated to suffer from Alzheimer’s disease (AD) while 121,499 deaths from the same pathology were reported in 2019 (Wiley, 2021). Centers for disease control and prevention (CDC) mention that by 2050 13.8 million US. adults aged ≥65 years are expected to suffer from AD (Taylor et al., 2017). Notably, these numbers refer to only one degenerative disease. Moreover, in many of these conditions such as AD, the only available treatment options are symptomatic and do not address the underlying cause of the disease (Breijyeh and Karaman, 2020). Thus, gene therapy for the treatment of CNS diseases holds the potential to bring promising results.

Although many approaches to deliver RNA molecules that do not contain viral vectors exist such as chemically modified antisense oligonucleotides (ASO), N-acetylgalactosamine (GalNAc) ligand-modified short interfering RNA (siRNA) conjugates (Kulkarni et al., 2021) or polymeric delivery platforms (Wahane et al., 2020) the role of non-viral, lipid vectors in the development of advanced therapeutic medicinal products for gene therapy is of great importance. In addition to protecting the nucleic acid from degradation, these platforms can be engineered to target the brain and ensure intracellular release through endosomal escape mechanisms in some cases. In this mini-review, we will discuss the approaches that are utilized for the development of lipid gene delivery nanoplatforms that target CNS and the perspectives of this technology in the treatment of brain diseases.

## Lipid-based nanoparticles

**FIGURE 1:**
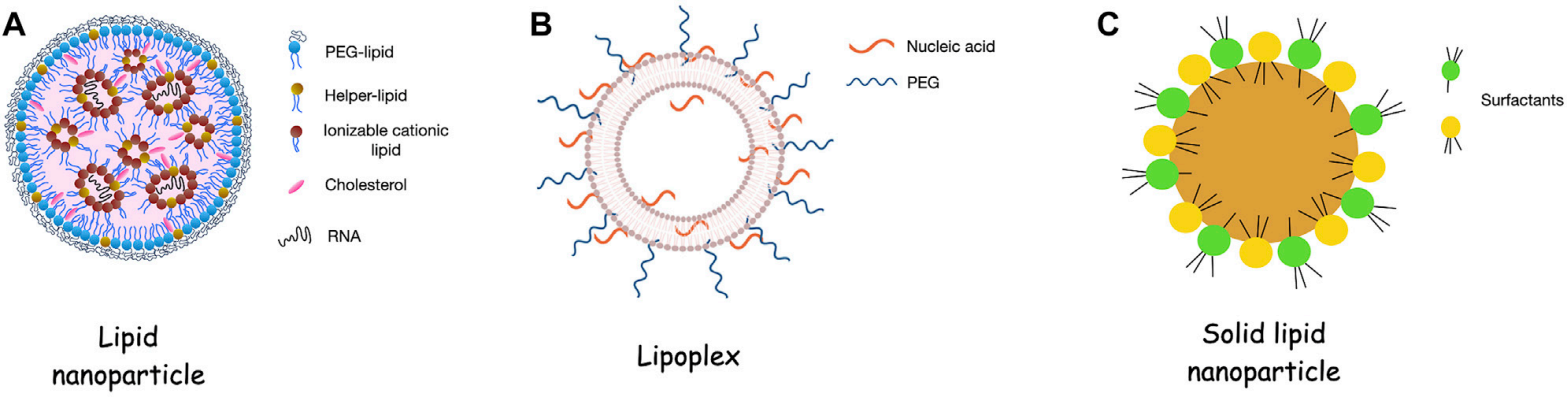
Consistency of lipidic nanoparticles for RNA delivery. **(A)**. Lipid nanoparticles (LNPs) contain an ionizable cationic lipid that allows the encapsulation of the RNA molecules into the internal aqueous cavities of the platform. Addition of PEG-lipid, helper lipid and cholesterol contribute in physicochemical and biological stability of the LNPs. **(B)**. Lipoplexes are liposomes that contain cationic lipids and are conjugated with nucleic acids i.e., RNA. IN this case the nucleic acids are mainly in the external surface of the systems leading which indicates that the RNA is more exposed to the environment than in the case of the LNPs. **(C)**. Solid lipid nanoparticles are formed by the lipid core of solid lipids and the amphiphilic surfactants that decorate the surface of the particles. Figure 1B adapted from Pereira et al. (2021).

Lipid-based nanoparticles are the most well-studied non-viral platforms for the delivery of RNA molecules (Zhao and Huang, 2014; Meng et al., 2017). This broad category includes liposomes, lipoplexes, lipid nanoparticles and solid lipid nanoparticles, as presented in Figure 1. By virtue of their biochemical composition, lipidic nanoparticles provide bio-mimicking and bio-degradable platforms. As a result of their lipophilicity, they can penetrate more easily the BBB while the inclusion of cationic lipids stabilize the negatively charged RNA molecules via electrostatic interactions.

### Liposomes and lipoplexes

Liposomes are self-assembled pseudospherical vesicles in which the lipid bilayers surround the aqueous core. Liposomes have been widely and for a long time used as drug delivery systems for the treatment of a plethora of diseases. Indeed, more than 15 liposomal drugs are currently on the market (Large et al., 2021). However, their application in encapsulating nucleic acids is in the pre-clinical or early clinical phase.

Liposomes can be categorized by their size, charge, key surface molecules or surface modifications to modulate circulation time and avoid detection and elimination by the immune system. The development of liposomes for gene therapy often involves the use of cationic lipids which interact electrostatically with the negatively charged genetic material (Mishra et al., 2022). The complexation of nucleic acids with cationic lipids leads to the formation of the lipoplexes in which nucleic acids are either on the external surface of the structure or in the internal cavity (Pereira et al., 2021). The most commonly used cationic lipids are 2,3-Dioleoyloxy-propyl)-trimethylammonium-chloride (DOTAP) (Lechanteur et al., 2018; Hattori et al., 2019), di-O-octadecenyl-3-trimethylammonium propane (DOTMA) and dimethyldioctadecylammonium bromide DDA (Putzke et al., 2020).

Concerning the central nervous system, certain liposomes/lipoplexes experiments have proved efficacious. Recently, Bender and others developed DOTAP:Cholesterol 1:1 liposomes that were incubated with small interfering RNA (siRNA) that decrease the amount of neuronal cellular prion protein (PrP^c^) and rabies virus glycoprotein fragment (RVG-9r). As it is presented in the literature, RVG selectively interacts with the nicotinic achetilocholine receptor on the surface of the neuronal cells while the addition of a positively charged arginine residue at the carboxyl terminus of RVG (9r modification) allows the electrostatic interaction of the modified peptide and the siRNA chains (Kumar et al., 2007). Indeed, their formulations can effectively bypass the BBB and prolong the lifetime of prion-infected mice. The targeting to the brain cells was successful due to the RVG-9r protein while the therapeutic effect suggests that the siRNA successfully knockdown the PrP^c^ expression (Zabel, 2013; Bender et al., 2016, 2019). Since many neurodegenerative diseases such as Alzheimer’s, Parkinson’s or Huntington’s pathology, belong to the category of prion diseases, those results could bring great promise for their treatment. In another study, DOTAP: Cholesterol siRNA-liposomes associated with transferrin that were topically administrated in mouse brain proved to have high efficiency in downregulation of pathological genes while having limited toxicity (Cardoso et al., 2008). Finally, Yuan et al. show that siRNA (siGOLPH3) loaded cationic liposomes incorporated with angiopep2GOLPH3 ligand for LRP-1 receptor that is expressed in human BBB and glioma cells, protein provide could result in a potential treatment of glioma (Yuan et al., 2018).

Furthermore, different routes of administration can improve the brain delivery efficiency of liposomes. Dhaliwal et al. chose to administer their mRNA lipoplexes intranasally. The liposomes were loaded with the mRNA, labeled with GFP-mRNA or luciferase-mRNA to evaluate the transfection efficiency of the formulations (Dhaliwal et al., 2020). The results proved that the expression of the mRNA was higher when the liposomal vesicles were utilized to vehiculate the mRNA than when the mRNA was administered in naked form. In comparison with the administration of the naked mRNA. Interestingly, Hu and others showed that intranasal administration of core-cell lipoplexes could effectively transfer the siRNA (Hu et al., 2022).

### Lipid nanoparticles

Disadvantages associated with the use of cationic lipids in liposomes and lipoplexes such as rapid clearance from the reticuloendothelial system (RES) due to opsonization and activation of the complement system and toxicity issues connected with cell apoptotic mechanisms, enhanced reactive oxygen species (ROS) levels, have prompted the development of the ionizable cationic lipids and the lipid nanoparticles (LNPs) (Filion and Phillips, 1997; Knudsen et al., 2015; Cui et al., 2018; Liu et al., 2020; Kulkarni et al., 2021). Cationic lipids that are positively charged at about pH 4, but that are electroneutral at physiological pH are key components of LNPs (Tenchov et al., 2021). Consequently, they provide a safer solution as advanced delivery platforms. Their safety is well-established due to the recent approval and massive administration of Pfizer/BioNTech and Moderna mRNA vaccines (Polack et al., 2020; Baden et al., 2021). However, only a few studies evaluate the safety and efficiency of LNP-RNA platforms intended for therapy in the CNS. To the best of our knowledge, the first research article to address this particular question was published in 2013. In this study, Rugta et al. provide evidence that when their siRNA-LNPs were administrated within mice cortex or via intracerebroventricular injection a good rate of uptake by neuronal cells and reduction of associated protein levels was observed. Furthermore, no toxicity to neurons was observed (Rungta et al., 2013). More recently, two separate research groups from Israel and Denmark have studied the potential role of RNA interference-LNPs in the treatment of glioma. The former, developed Dlin-MC3-DMA, DSPC, Chol, DMG-PEG, and DSPE-PEG LNPs decorated with hyaluronan, which is a CD44 ligand (Cohen et al., 2015). The latter, chose a more complex formulation that externally has a slight negative charge due to a PEGylated cleavable lipopeptide. Metalloproteinases present in the tumor microenvironment are responsible for the cleavage of the PEGylated lipoprotein leading to positively charged LNPs that subsequently endocytosed. Thus, intracellular release of the siRNA is achieved (Bruun et al., 2015). Both groups resulted in good toxicological results and a high knockdown percentage of the targeted mRNA after *in vitro* experiments and topical administration in mice. Tanaka et al. developed LNPs that contained SS-cleavable proton-activated lipid-like materials as the ionizable elements. These synthetic lipids provide a neutral charge of the platform at physiological pH values while they are protonated at the low endosomal pH. Similarly with the work of Bruun et al. (2015), such a phenomenon leads to the protection of the RNA molecule when administrated *in vivo* and the intracellular release of this sensitive bio-molecule (Tanaka et al., 2018). Finally, Khare et al., recently showed that LNPs with C_12-200_ lipidoid, which is a syntetic lipid-like molecule that displays tertiary amines in the headgroups, could provide a safer approach for RNA delivery into the neurons than other platforms that contain lipofectamine [mixture of 2,3-dioleoyloxy-N- [2 (sperminecarboxamido)ethyl]-N,N-dimethyl-1-propaniminium trifluoroacetate (DOSPA) and 1,2-Dioleoyl-sn-glycerophosphoethanolamine (DOPE)]. The platform offers favourable loading capacity, displays efficient cellular uptake and low *in vitro* cytotoxicity in human cortical cell lines (Khare et al., 2021).

### Solid lipid nanoparticles

Solid lipid nanoparticles (SLNs) are another category of lipidic nanoparticles. They from liposomes in that SLNs do not have an aqueous central cavity but a solid lipid:surfactant one (Scioli Montoto et al., 2020). Depending on their composition, size and charge SLNs present unique physicochemical properties (Naseri et al., 2015).

Concerning their role in the protection and delivery of nucleic acids, SLNs have not been widely studied, maybe due to their morphological characteristics. Since nucleic acids are hydrophilic molecules, their interaction with the SLN could take place only on the surface of the delivery platforms electrostatically. However, electrostatic interactions are not particularly stable and could easily break under environmental pressure (Wojnarowska et al., 2018). Nevertheless, an interesting study was performed by Rassu et al. They stabilized BACE-1 siRNA negatively charged molecules, that could have a valuable role in the treatment of Alzheimer’s disease, with RVG-9R, which was positively charged. Afterward, the siRNA-RVG-9R complex solution was mixed with triglycerides, poly (vinyl alcohol) and chitosan for the formation of a double emulsion that contains the SLNs. The chitosan coated SNPs presented higher *in vitro* mucoadhesiveness and permeability properties than the non-coated SNPs (Rassu et al., 2017). Another study utilized two different siRNA molecules that knockdown the human c-Met, which is found in glioma to deliver them by SLN. The siRNA molecules were first conjugated with polyethylene glycol (PEG) via a disulfide bond. The SLN-siRNA-PEG formulations displayed accumulation in the brain tumor, no systemic toxicity as well as suppression of the tumor growth after intravenous administration in the glioblastoma xenograft tumor model (Jin et al., 2011).

## Discussion

Today, the treatment of brain malignancies such as glioblastoma or neurodegenerative diseases still remains an elusive (Mullard, 2021; Sun and Roy, 2021). Gene therapy is a promising approach to developing new medicinal products for a plethora of different diseases (Sudhakar and Richardson, 2019; Parambi et al., 2022). For instance, the small interfering RNAs can downregulate the expression of pathological proteins that are present in diseases. Such pharmacological action is not possible with other therapeutic approaches (i.e., by small drugs or proteins). Viral vectors have been the gold standard of brain gene delivery in the past years as they can easily pass the BBB, they are taken up by the brain cells and transfer their genes into the cell nucleus (Lundstrom, 2018). Although the viruses that are utilized are either attenuated or non-replicating, concerns about the safety of these vectors exist in terms of immunogenicity and mutagenicity.

The BBB remains one of the main obstacles for the lipidic delivery systems. Three pathways responsible for the lipidic vesicles by-pass through the BBB are mentioned in the literature: 1) adsorptive-mediated transcytosis (AMT), 2) receptor-mediated transcytosis (RMT), and 3) carrier-mediated transcytosis (CMT) (Juhairiyah and de Lange, 2021). Concerning AMT, cationic vesicles interact electrostatically with the endothelial cells of the BBB. However, the cationic charge leads to toxicity effects and fast inactivation of the particles via opsonization processes. The decoration of the lipidic nanoparticles with accessory molecules such as peptides or antibodies is a common procedure. Transferrin (Kong et al., 2020), glucose or mannose and their derivatives (Qu et al., 2014; Arora et al., 2020), vitamin C (Peng et al., 2018) and glutathione (Salem et al., 2015; Trapani et al., 2021) are some of these accessory molecules. Another effective way to avoid the BBB via non-invasive administration is the nose-to-brain pathway. Although the exact mechanism of action of nose-to-brain delivery is not clear, transfer through olfactory nerves or trigeminal nerves seems to be the most common way after intranasal administration. For the former, the nanoparticles paracellularly pass through the gaps of olfactory cells, finally reaching the subarachnoid space (Crowe et al., 2018). Indeed, Godfrey and others found that their intranasally administrated nanoplatforms were located in the olfactory bulb only 5 min after the administration (Godfrey et al., 2018). On the other hand, the transport through the trigeminal nerves seems to be less common but possible both by intra- or extra-cellular routes (Cunha et al., 2017; Bourganis et al., 2018). However, some consideration still exists of whether today, nose-to-brain administration of nanovesicles increase the effectiveness of active molecules (Feng et al., 2018).

The majority of recent and current studies related to the delivery of small molecules or bio-molecules in the CNS with the aid of nanotechnology have focused *on* liposomal nanovesicles. However, other platforms, such as LNPs may be better choices for the transport of genetic material in the brain due to the better encapsulation of a sensitive RNA cargo. For instance, although many of the studies mentioned above indicate that LNPs containing RNA molecules could play an important role in the treatment of CNS diseases, all of them used a patient unfriendly route of administration and more work is necessary for the development of platforms that are not only safe and efficacious but also not demanding for the patient. Moreover, *in vivo* toxicity studies after repeated administration is necessary to verify the safety of the platform as in many cases the therapeutic regimen would entail multiple, rather than single, dose protocol. Nevertheless, lipidic nanoparticles that carry RNAs hold great promise as advanced therapeutic medicinal products that could provide effective results in the treatment of neurodegenerative disorders, such as Alzheimer’s disease, or brain tumors like glioblastoma.
